# Population genomics provide insight into ancestral relationships and diversity of the feral horses of Theodore Roosevelt National Park

**DOI:** 10.1002/ece3.11197

**Published:** 2024-04-01

**Authors:** Melissa A. Thompson, Blake E. McCann, Turk Rhen, Rebecca Simmons

**Affiliations:** ^1^ Department of Biology University of North Dakota Grand Forks North Dakota USA; ^2^ Theodore Roosevelt National Park National Park Service Medora North Dakota USA

**Keywords:** Equus, feral horse, genetic drift, genomics, population

## Abstract

Theodore Roosevelt National Park (TRNP) manages a herd of feral horses (*Equus caballus*) which was present on the landscape prior to the establishment of the park. The population presents a unique scenario in that it has experienced fairly intensive and well‐documented management since the park's establishment, including herd size reductions, intentional introduction of diversity, and subsequent attempts to remove introduced lineages. This provides an interesting case study on the genetic effects of diverse evolutionary forces on an isolated feral population. To explore the effects of these forces and clarify the relationship of this feral herd with other horses, we used genome‐wide markers to examine the population structure of a combined dataset containing common established breeds. Using the Illumina Equine 70k BeadChip, we sampled SNPs across the genome for 118 TRNP horses and evaluated the inbreeding coefficient *f* and runs of homozygosity (RoH). To identify breed relationships, we compared 23 representative TRNP samples with 792 horses from 35 different breeds using genomic population structure analyses. Mean *f* of TRNP horses was 0.180, while the mean *f* for all other breeds in the dataset was 0.116 (SD 0.079). RoH analysis indicates that the TRNP population has experienced recent inbreeding in a timeframe consistent with their management. With Bayesian clustering, PCA, and maximum likelihood phylogeny, TRNP horses show genetic differentiation from other breeds, likely due to isolation, historical population bottlenecks, and genetic drift. However, maximum likelihood phylogeny places them with moderate confidence (76.8%) among draft breeds, which is consistent with the known history of breeds used on early North Dakota ranches and stallions subsequently introduced to the park herd. These findings will help resolve speculation about the origins of the herd and inform management decisions for the TRNP herd.

## Graphical abstract

We used SNP markers and population genomics analyses to identify relationships between a population of free‐roaming feral horses and other domestic horse breeds. The Theodore Roosevelt National Park (TRNP) horses show genetic differentiation from other breeds, likely due to isolation, historical population bottlenecks, and genetic drift, however, we identified draft horse breeds as most similar to the TRNP horses. This is consistent with the known history of herd management.

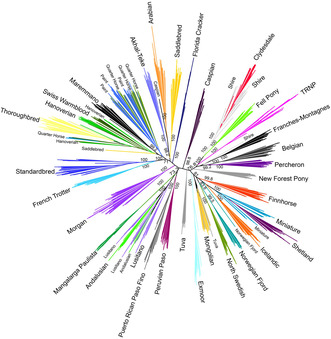

## 1 INTRODUCTION

Feral or free‐roaming horses (*Equus caballus*) can be found in many locations within the United States and in many other countries around the world, some with more recent or well‐documented origins than others. These populations are often isolated to varying extents and usually require some form of management. There is strong public interest in feral horses on public lands and concern about the genetic health of small populations. Here, we present a case study of a feral horse population which has undergone fairly intensive and well‐documented management since the 1940s and 1950s. This creates a unique study system to examine the effects of diverse evolutionary forces interacting with management actions upon the population genetics of a herd. There is also increasing public interest in the origin of this population, and a need for evidence‐based decisions when considering management strategies.

### 1.1 TRNP herd history

Free roaming horses existed in the badlands of southwestern North Dakota when Theodore Roosevelt National Park (TRNP) was established in 1947. At that time, many of the horses roaming within the boundary of the park were owned and branded by local ranchers. The horses had either escaped or were released to forage and reproduce on their own, so that ranchers could recapture the horses and their offspring for later use (McLaughlin, 1989). In 1954, the park began work on the task of erecting a perimeter fence for the reintroduction of bison (*Bison bison*); an effort was made to round up the horses, which at that time were considered trespass livestock, to return them to their owners. Approximately 125 horses and mules were captured of the estimated 200 head present within the park boundary, 99% of which bore brands as evidence of ownership (McLaughlin, 1989). Over the next decade there were unsuccessful efforts to remove all remaining horses; in the 1970s, park administration decided to maintain the horses as a “historic livestock display” or “living history demonstration”, reminiscent of the free‐roaming livestock that Theodore Roosevelt documented during his residency (Harmon, 1986).

Reports vary as to the number of horses left in TRNP after these removal attempts, with some suggesting that the remaining founder individuals were one gray stallion and two mares. The consensus among reports, however, indicates that there were only about 16 individuals present in 1965 (Harmon, 1986; McLaughlin, 1989). Every few years thereafter the park conducted roundups to control population size by removing a portion of the herd. Population size was initially selected as 35–60 individuals (National Park Service, 1978). A habitat use and forage analysis later recommended a population maximum of 90 individuals to prevent overgrazing of some forage species (Marlow et al., 1992). More recently, a population objective of 70–140 animals was suggested following a genetic analysis which found low effective population size (Cothran, 1992). Ten roundups were conducted from 1978 to 2013; each time the population was reduced by an average of 52% (TRNP records). Thus, the TRNP horse population has undergone 11 potential population bottleneck events. The herd has mostly been a closed population since the park perimeter was fenced. A few animals have likely entered the population over the years by mistake or intentional disposal by private persons, though all known trespass horses of the past 20 years have been removed (B. McCann, personal communications). In 1981 and 1982, several established TRNP stallions were removed as part of an attempt to augment the herd by introduction of “well‐bred” stallions. Six stallions were introduced at this time, including an Arabian, a Shire‐Paint cross, a Quarter Horse, and three feral stallions from a Wyoming herd. Each had varying levels of reproductive success within the population (McLaughlin, 1989). The Shire‐Paint cross stallion was reported to be highly successful: he maintained a large band of mares for almost a decade and was considered the most dominant stallion in the park. An estimated 15% of the population could be traced to this single stallion in 1991 (Cothran, 1992). Introduction of stallions was discontinued in favor of maintaining the historic type; in 1991 and 1997 attempts were made to remove the introduced stallions and some of their known offspring (TRNP records).

An oral history of the herd collected from TRNP employees and local ranchers in 1989 suggested that some of the horses which eluded capture originally were descended from “Indian type” horses of Spanish descent (McLaughlin, 1989). The horses used for ranch work in late nineteenth century North Dakota some 70 years prior to the park's establishment were often “Indian type” horses crossbred with other European breeds from the eastern US, Texas, Colorado, and Idaho (Crawford, 1931; Huidekoper, 1947; McLaughlin, 1989). Some have suggested that the TRNP herd is a unique population due to this potential association with the historical Spanish‐type horse; however, this assertion has not been substantiated with genetic evidence. A phenotypic evaluation based on physical conformation and coat colors found evidence of crossbreeding in all TRNP horses and the presence of Spanish‐type features, but recommended genetic evaluation for further resolution (Sponenberg, 1994).

Previous studies have estimated genetic diversity for the TRNP horses. Seven red blood cell antigen loci were tested in the 1990s to calculate genetic variability measures for the herd. These values were compared over a nine‐year period from 1991 to 2000, as well as compared to average values for domestic horse breeds and other feral horse populations. There were decreasing values for expected heterozygosity (*H*_e_) over the sampling period, with the final value falling below the average for both domestic and feral horses (*H*_e_ = 0.327, compared to 0.363 and 0.349 for domestic and feral populations, respectively). The report also found a lower effective number of alleles (*A*_e_) than the average for both domestic and feral populations (Cothran, 2000). All reported allele variants were previously described in domestic breeds; no unique alleles were found in the TRNP population (Cothran, 1992). A later analysis of mitochondrial DNA (mtDNA) and 12 short tandem repeat (STR) loci from the present‐day TRNP herd was conducted by Ovchinnikov et al. (2018). Values of *H*_e_ and *A*_e_ found for the TRNP herd were again lower than the average for other feral herds and most domestic breeds. Three mtDNA haplotypes were found and fully sequenced, none of which were exact genome matches to any published sequences in GenBank. Two of the mitochondrial genomes belonged to the same haplogroup and were most similar to an American Paint Horse sequence; the other belonged to a second haplogroup and had no close match to published sequences. However, the control region sequences of both haplogroups had matches to a wide variety of breeds with a global distribution. This suggests that at least two different populations or maternal sources contributed to the genetic diversity of the TRNP herd. The STR analysis was inconclusive in determining the ancestry of the park horses and showed TRNP horses as distinct from other breeds (Ovchinnikov et al., 2018).

Genomic approaches can be used to gain insights into population structure, relatedness, and genetic diversity for TRNP horses. The development of a horse reference genome allows for a genome‐wide analysis, using tens of thousands of single nucleotide polymorphisms (SNPs) (Wade et al., 2009). Genome‐wide SNPs have been used in many horse breeds to measure genetic diversity, identify regions of diversifying selection, and make inferences about the origins of breeds (Cosgrove et al., 2020; Gurgul et al., 2019; Petersen, Mickelson, Cothran, et al., 2013). Prior studies of domestic breeds and feral herds suggest that population genetics can be a useful tool for investigating breed associations and the potential origins of feral herds such as the horses of TRNP.

Here, we use genome‐wide SNP genotypes to examine the relationship of the TRNP horses to established horse breeds. Based on the known history of the herd, we hypothesized that TRNP horses would be most genetically similar to common ranch horses in the USA today (Quarter Horses and American Paint Horses), Shires, or Spanish‐type breeds, but that they would primarily appear as a distinct population apart from any one breed. We also hypothesized that confinement and management actions have contributed to recent loss in genetic diversity within the TRNP herd. We found that TRNP horses are distinct from other breeds, likely due to isolation, population bottlenecks occurring as part of herd management, and genetic drift. For similar breeds, we found that these horses are most closely allied with draft breeds, particularly the Shire, which were historically associated with surrounding ranches and documented stallion introductions in the park, but not notably related to Quarter Horses, Paints, or Spanish‐type breeds.

## 2 METHODS

### 2.1 Sample collection

Hair samples were collected during a regularly‐scheduled roundup in 2013. From these, we selected samples of 87 horses which had been re‐released into the park, including almost all adult mares and reproductive band stallions. In 2017, additional tissue samples were collected as part of management activities via biopsy dart from 12 individuals that had evaded the 2013 capture. With the addition of these individuals our sample set represented an approximate census of the herd as it was at the end of 2013 (99/107 = 92.5%). In 2020 we collected an additional 18 tissue samples from young mares born post‐roundup by biopsy dart. Our full dataset had 118 samples (91 mares, 27 stallions), including a sample from one more young mare. This includes 85% (94/111) of the adult individuals in the herd as of spring 2022 and represents 98% (177/181) of the herd when including offspring of sampled individuals.

### 2.2 DNA extraction, genotyping, and sample selection

Genomic DNA was extracted from hair follicle and tissue samples by the Animal Genetics Laboratory at Texas A&M University using Gentra Puregene Tissue kits (Qiagen) following manufacturer's protocols. Individuals were then genotyped at Neogen Genomics Laboratory (Lincoln, NE) for over 70k SNPs located evenly across the horse genome using the Illumina Equine GGP 70 k BeadChip. We combined the resulting genotypes with a dataset of 792 horses from 35 different breeds which had been genotyped using the Illumina Equine GGP 50k BeadChip (Petersen, Mickelson, Rendahl, et al., 2013). Mean sample size for breeds in that dataset was 22.63 individuals (Table A1); to prevent a comparatively large number of TRNP samples from skewing the principal components analysis (PCA), we selected a representative subset of 23 TRNP individuals for our final dataset. Using pedigree data determined by genetic testing and family band association records going back to the 1980s, we identified first degree relatives and excluded the younger individual (i.e., offspring) to reduce bias due to relatedness, which left 32 individuals. We then performed a random selection to reduce the dataset to the final 23 individuals, and manually checked to confirm that this subset contained individuals from both geographic regions within the park.

### 2.3 Data pruning

We performed quality control filtering using SNP & Variation Suite v8.9.0 (SVS) (Golden Helix, Inc., Bozeman, MT, www.goldenhelix.com), with the methods used by Petersen, Mickelson, Cothran, et al. (2013). We first removed markers with call rate ≤0.95, and then samples with call rate ≤0.95. All 23 TRNP samples were above the threshold and were retained. This eliminated any markers which were not included in both the 70k and 50k genotyping arrays. We next removed SNPs with a minor allele frequency (MAF) of 0.05 or less. We mapped the remaining SNPs to EquCab3.0 (www.ncbi.nlm.nih.gov/assembly/GCF_002863925.1/) using SVS and filtered to include only autosomal loci. This resulted in a final set of 815 samples and 38,786 SNPs. We further filtered the dataset for linkage disequilibrium (LD) using a window size of 50 and an increment of 5, with an LD threshold of *r* ^2^ = .5. After LD filtering, this second version of the dataset retained 815 samples and 28,505 SNPs.

### 2.4 Among‐breed relationships

To assess the current relationship of the TRNP horses to other breeds, we conducted a Principal Components Analysis (PCA) on the dataset pruned for MAF and call rate, as per Petersen, Mickelson, Cothran, et al. (2013). We computed the principal components in SVS using an additive model with the option selected to normalize each marker's data by its standard deviation. We then plotted the first three principal components against each other to visualize relationships among individuals of different breeds and the TRNP horses. We also calculated pairwise values of Wright's fixation index (*F*_ST_) between all breeds and TRNP horses in SVS using the LD pruned dataset.

### 2.5 Phylogenetic analysis

We conducted a phylogenetic analysis to depict evolutionary relationships among the populations. We converted the LD pruned dataset to Phylip format using vcf2phylip (Ortiz, 2019). We included all breed samples from the Petersen, Mickelson, Cothran, et al., 2013 dataset along with the same 23 TRNP samples used for the PCA and *F*_ST_ analyses. All phylogenetic analyses were performed via the CIPRES Science Gateway v3.3 (Miller et al., 2010). We converted the SNP dataset to a FASTA file using NCL converter v2.1, which maintained the original nucleotide information (Lewis, 2003). The final matrix included 815 individuals sampled for 28,501 aligned nucleotides. We aligned the resulting data using ClustalW v2.1 with standard parameters (Thompson et al., 1994), and used RAxML v8.2.12 (Stamatakis, 2014) to construct the phylogeny for these samples, with 1000 bootstrap replicates. Bootstrap values were calculated using a majority rule consensus tree with Consense (Felsenstein 1986–2008). Only bootstrap values of ≥70% are reported. We visualized the resulting phylogeny and edited the appearance of the tree using FigTree v1.4.4 (http://tree.bio.ed.ac.uk/software/figtree/).

### 2.6 Bayesian cluster analysis

To estimate ancestral clusters and common heritage, we used the program ADMIXTURE on the LD pruned dataset (Alexander et al., 2009). ADMIXTURE employs model‐based ancestry estimation to assign individuals to clusters with similar ancestry based on genotypes. For each individual sample ADMIXTURE returns the proportion of their genome that can be assigned to each ancestral cluster based on allele frequencies. Since there were 36 breeds including the TRNP samples in the dataset, we ran the program for *K* = 1 through *K* = 36, *K* being the assumed number of ancestral populations. To determine the value of *K* that created the most accurate model to describe the dataset we followed ADMIXTURE instructions and compared the program's values for cross‐validation (CV) error at each value of *K*, using the default setting of 5‐fold CV and selecting the lowest resulting value.

### 2.7 Estimates of inbreeding

To assess inbreeding and the effects of historical population management, we conducted two measures of estimating inbreeding levels. We calculated the individual inbreeding coefficient (*f*) for all 118 TRNP samples and each breed sample in the LD pruned dataset using SVS. Another approach to evaluating inbreeding or relatedness by descent is to identify runs of homozygosity (ROH) within the genome. These homozygous‐by‐descent (HBD) segments are created when an individual inherits two copies of the same stretch of chromosome from a common ancestor (Ceballos et al., 2018; Peripolli et al., 2016). We used the R package RZooRoH to identify ROHs and model the generational age of common ancestors based on segment length (Bertrand et al., 2019). RZooRoH uses hidden Markov models to relate the length of HBD segments to the age of the segments, as a more recent common ancestor will have had fewer opportunities for recombination of the homozygous segment. To evaluate the state of the TRNP herd we included all 118 TRNP samples, did not prune for LD, and used the RZooRoH default model. We used Vortex10 (Lacy & Pollak, 2021) to calculate the generation time of the TRNP herd using historical records of demographic survival and mortality rates. The resulting generation time of 10.48 years closely matched the 10 years previously reported for feral horses (National Research Council, 2013). We also selected several other breeds with known population history for comparison.

### 2.8 TRNP population structure

To examine subpopulation structure within the TRNP herd, we prepared a dataset of all 118 samples pruned by marker call rate ≤0.95, sample call rate ≤0.95, MAF ≤0.05, and autosomes only. We then conducted a PCA of this dataset in SVS as above. We assigned samples to geographic regions based on observational data of spatial use by each family band of horses. The South Unit of the park was partitioned into two geographic regions by an intermittent stream: the categories were “North of Paddock Creek (NoPC)”, “South of Paddock Creek (SoPC)”, or individuals of mixed parentage resulting from observed dispersal.

## 3 RESULTS

### 3.1 Among‐breed relationships

**FIGURE 1:**
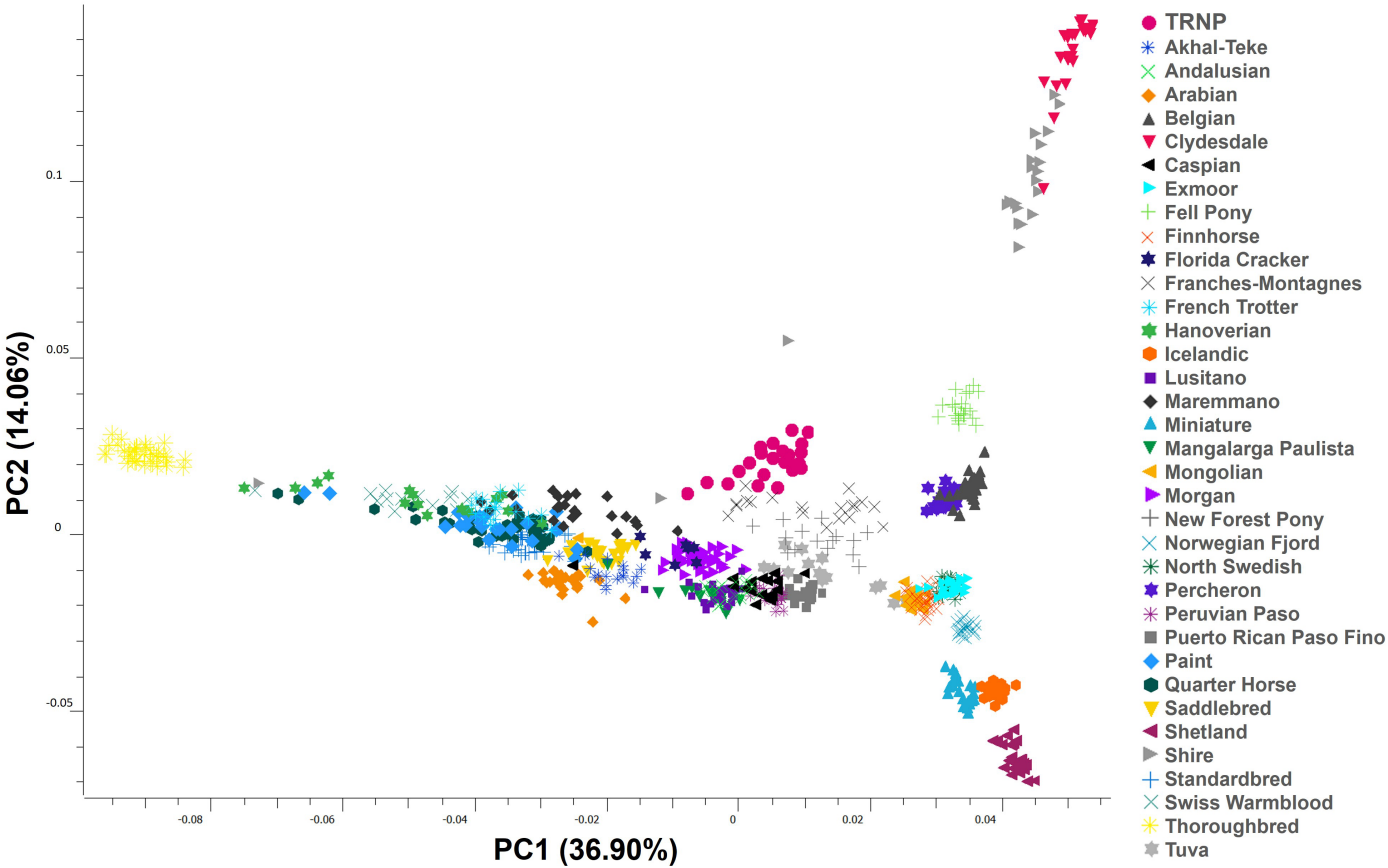
Principal components analysis of genetic variation among horse breeds and feral horses from TRNP, showing one plane of the cloud of points (PC1 by PC2). Points represent individuals. PC1 captures 36.90% of the variation in the dataset. PC2 captures 14.06% of the variation. TRNP horses fall into a group in the center of the plot, indicated by pink circles.

**FIGURE 2:**
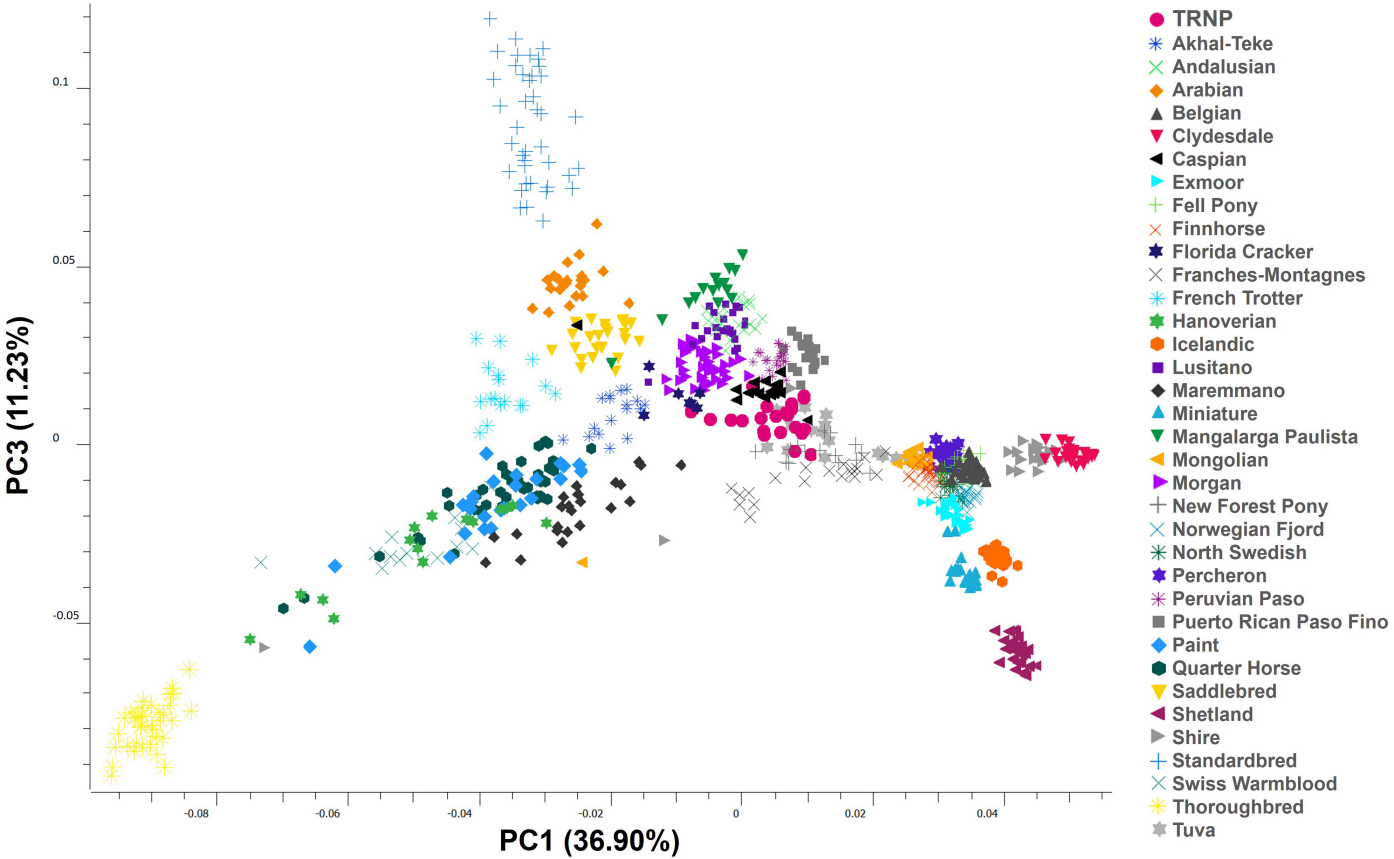
Principal components analysis of genetic variation among horse breeds and feral horses from TRNP, showing the same cloud of points from another plane (PC1 by PC3). Points represent individuals. PC3 captures 11.23% of the variation in the dataset. TRNP horses fall into a group in the center of the plot, indicated by pink circles.

The first three principal components from our PCA account for 62.19% of total variation in the dataset. The first principal component (PC1) explains 36.90% of the variance in the dataset. The second principal component (PC2) explains 14.06% of the variance, with a more gradual decline in explanatory value from PC2 to PC10 (Table A2). On a plot of PC1 by PC2, the breeds follow the pattern described by Petersen, Mickelson, Cothran, et al. (2013). TRNP horses fall near the center of the plot (Figure 1). The TRNP cluster overlaps on PC1 with such breeds as Morgan, Lusitano, Mangalarga Paulista, Andalusian, Franches‐Montagnes, New Forest Pony, Peruvian Paso, Tuva, Caspian, and Puerto Rican Paso Fino. On PC2, the TRNP horses are separated from these breeds in the direction of the draft horses such as Shire, Clydesdale, and Fell Pony. When looking at PC3 on the plot of PC1 by PC3 there is more overlap of the TRNP horses with the Tuva, New Forest Pony, and Caspian, as well as with the Akhal‐Teke and French Trotter (Figure 2).

Mean pairwise *F*_ST_ value among breeds was 0.108, with a minimum value of 0.002 between Paint and Quarter Horse and a maximum value of 0.273 between Clydesdale and Mangalarga Paulista (Table A3). *F*_ST_ values between the TRNP horses and other breeds ranged from 0.104 to 0.217. Breeds with the lowest *F*_ST_ values in comparison with the TRNP horses were the Tuva (0.104), New Forest Pony (0.107), Quarter Horse (0.108), Paint Horse (0.108), Mongolian horse (0.111), Maremmano (0.115), and the Morgan (0.115). The breeds with the highest values were the Mangalarga Paulista (0.217), Clydesdale (0.213), Exmoor (0.203), Shetland (0.184), and Thoroughbred (0.180).

### 3.2 Phylogenetic results

**FIGURE 3:**
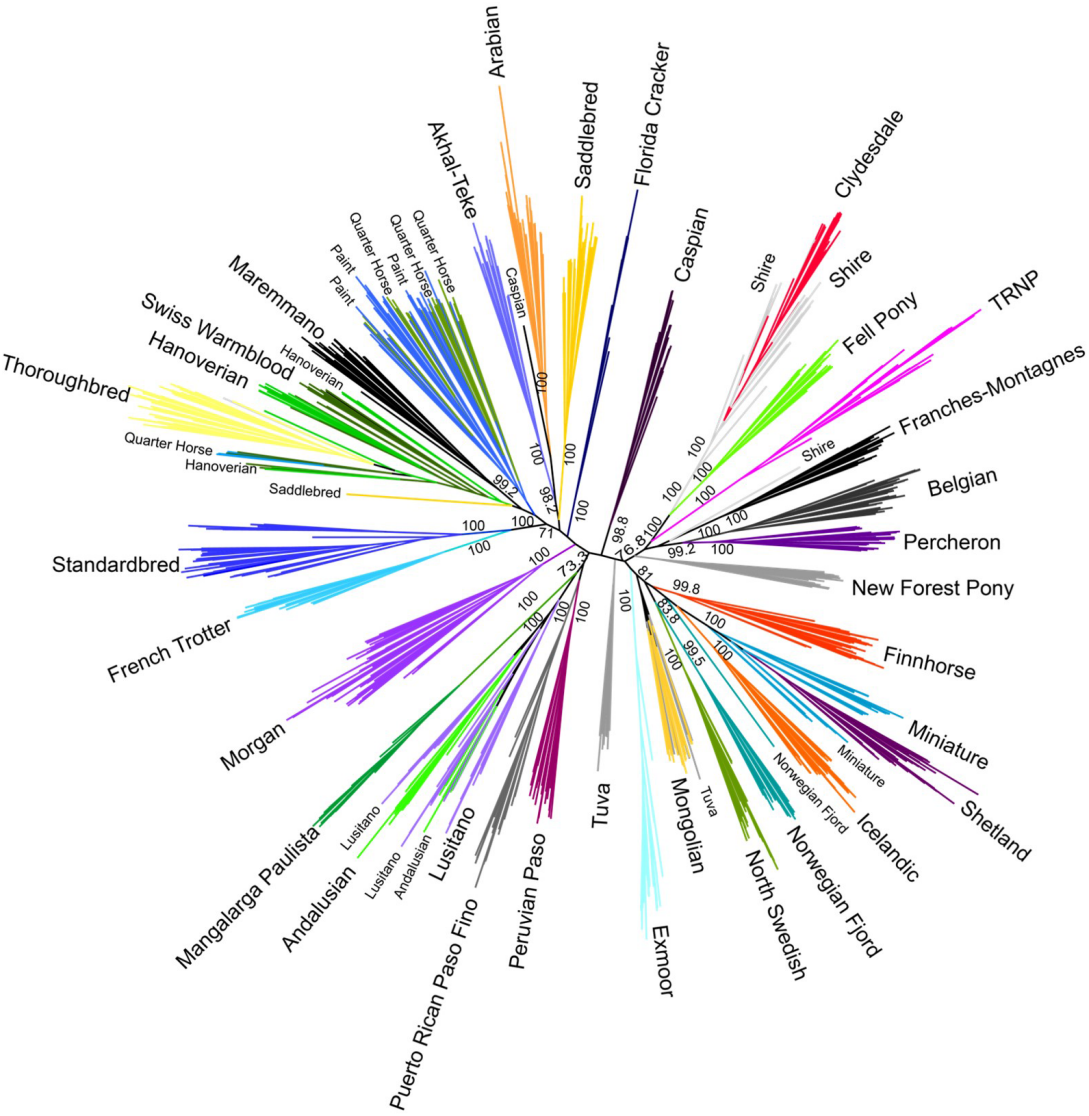
Maximum likelihood tree with bootstrap values for horse breeds, including TRNP horses. Only bootstrap values with confidence of 70% and higher are given. TRNP horses were found to be monophyletic and were placed among draft breeds with 76.8% confidence, diverging from the branch that contains Shires, Clydesdales, and Fell Ponies.

The results of the maximum likelihood analysis revealed a star phylogeny pattern with short internal branches and long external branches, typical of a lineage which has undergone rapid differentiation (Figure 3). Samples with the same breed assignment were placed mostly into their own respective clades. External nodes had high confidence bootstrap values, but many of the basal branches joining breeds were not well supported (<70% bootstrap values). TRNP horses were found to be monophyletic with strong support (100% bootstrap support) and were placed with moderate support (76.8% bootstrap support) with the Shire, Clydesdale, and Fell Pony, among other draft‐type breeds such as the Percheron, Belgian, and Franches‐Montagnes. The next nearest branches included the pony‐type breeds (e.g., Finnhorse, Miniature, Shetland); this clade had higher support (81% bootstrap support). Spanish‐type breeds (e.g., Andalusian, Peruvian Paso, Mangalarga Paulista) comprise a clade that is separate from TRNP horses with moderate support (73.3%).

### 3.3 Bayesian clustering analysis

**FIGURE 4:**
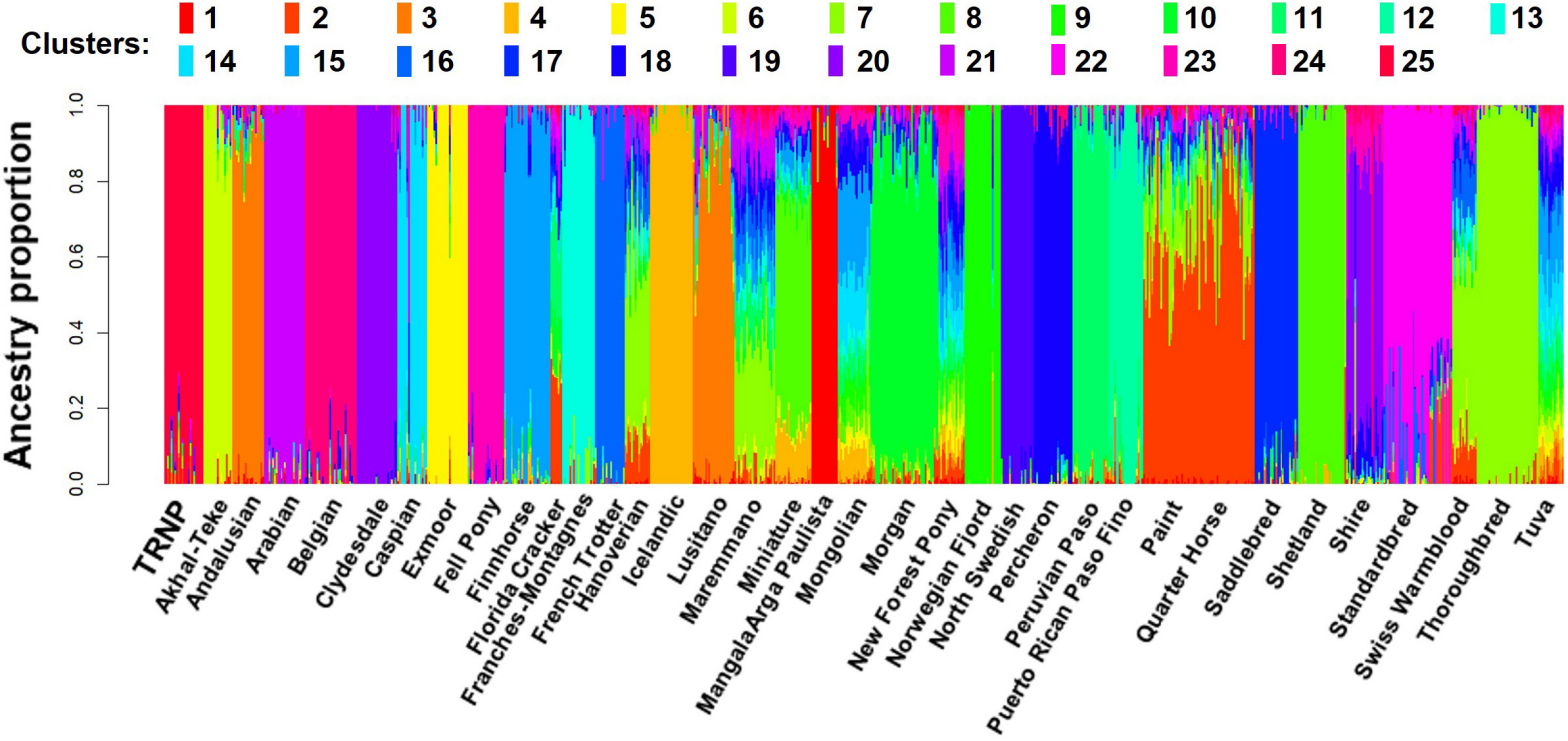
Ancestry estimation using ADMIXTURE modeling. The number of ancestral populations (clusters) *K* = 25 was chosen based on ADMIXTURE's CV error calculation. Vertical lines represent individuals, with colors representing the proportion of their genome attributed to each ancestral cluster. The TRNP horses make up their own red cluster (leftmost) with minimal shared ancestry from other clusters.

**FIGURE A1:**
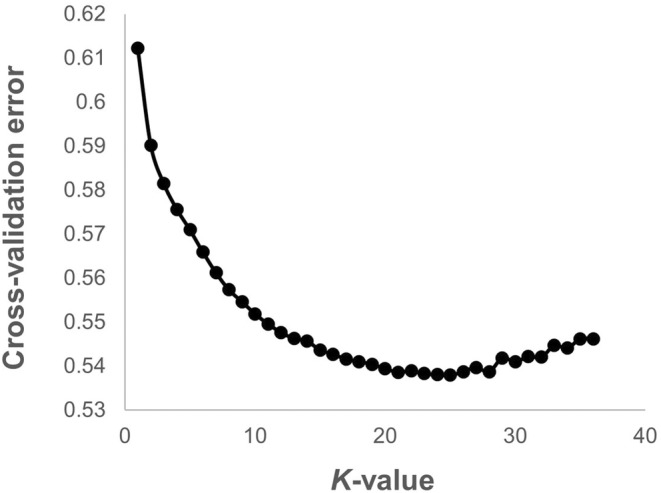
Cross‐validation error for each value of *K*, calculated by ADMIXTURE. The most likely value of *K* was chosen by the lowest error value.

The lowest CV error returned by ADMIXTURE was observed at *K* = 25 distinct populations, though CV error values of *K* in the 21–28 range were of similarly low values (Figure A1). At *K* = 25 each TRNP horse was grouped by a majority of their genome into the same cluster with minimal assignment to other clusters (Figure 4). The proportion of TRNP genomes assigned to this cluster ranged from 0.703 to 0.999, with a mean of 0.952. No individuals from other breeds had notable assignment to the TRNP horse cluster. Four TRNP individuals had a proportion of 0.039–0.075 of their genomes assigned to another cluster which included high genome proportions from Quarter Horse, Paint, and Florida Cracker individuals, and a 0.021–0.033 proportion assigned to a cluster including Thoroughbred, Hanoverian, Shire, Quarter Horse, Paint, Swiss Warmblood, and Maremmano individuals. Two TRNP individuals had 0.010–0.031 of their genome assigned to each of three clusters including Andalusian, Lusitano, Percheron, Shire, and Clydesdale individuals. The individuals from other breeds that had the highest proportion of assignment with the TRNP cluster (range 0.032–0.054) were a Swiss Warmblood, Hanoverian, Morgan, Saddlebred, two Paints, Maremmano, Quarter Horse, Puerto Rican Paso Fino, and a New Forest Pony.

**FIGURE A2:**
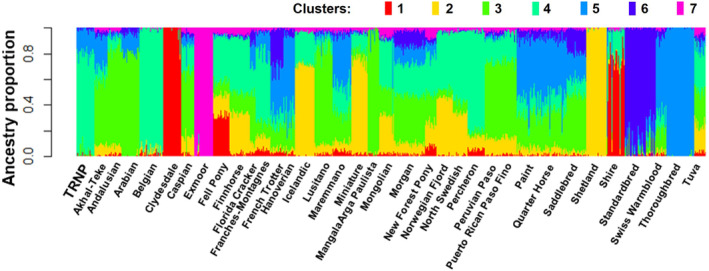
Ancestry estimation using ADMIXTURE modeling with the value of *K* = 7, the lowest value of K for which the TRNP horses are primarily assigned to a cluster that includes other breeds. Vertical lines represent individuals, with colors representing the proportion of their genome attributed to each ancestral cluster. The TRNP horses are assigned to a cluster that also includes individuals from many breeds of draft type, including Belgian, Percheron, Franches‐Montagnes, North Swedish Horse, Norwegian Fjord, Finnhorse, Mongolian, Tuva, and New Forest Pony. The Shire and Clydesdale make up their own cluster at *K* = 7.

**FIGURE A3:**
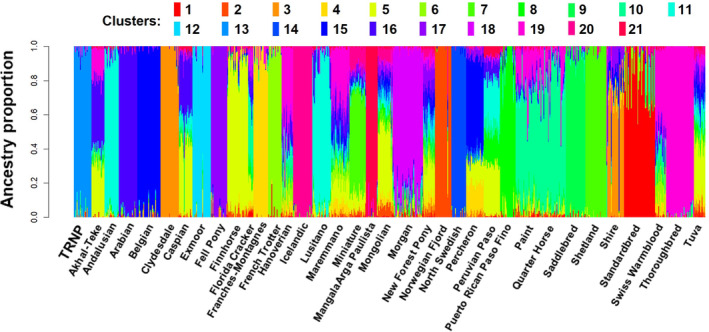
Ancestry estimation using ADMIXTURE modeling with the value of *K* = 21. Vertical lines represent individuals, with colors representing the proportion of their genome attributed to each ancestral cluster. At *K* = 21 the CV error is only slightly higher than the lowest value of *K* = 25. At *K* = 21, the TRNP horses are still assigned to their own cluster, which no other individuals share.

In the *K* = 21–28 range of low CV errors the TRNP horses clustered similarly to the *K* = 25 results (Figure A3). From *K* = 20 through *K* = 8 the TRNP horses were still assigned to their own cluster. At *K* = 7, the TRNP horse genomes were assigned by 0.621–0.850 to a cluster that included high proportions for other individuals of many different breeds of draft type, such as Belgian, Percheron, Franches‐Montagnes, and North Swedish Horse (Figure A2). At such a low number of ancestral populations most of the clusters separated into general groups of draft breeds (the Shire and Clydesdale formed their own cluster), pony breeds, Spanish, and Arabian breeds, or warmblood breeds, reflective of PCA and phylogenetic relationships.

**FIGURE A4:**
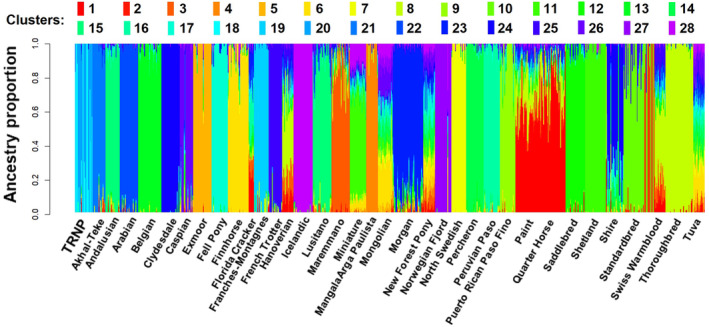
Ancestry estimation using ADMIXTURE modeling with the value of *K* = 28. Vertical lines represent individuals, with colors representing the proportion of their genome attributed to each ancestral cluster. At *K* = 28 the CV error is only slightly higher than the lowest value of *K* = 25. At *K* = 28, the TRNP horses are split into two clusters, still unshared with any other breed.

**FIGURE A5:**
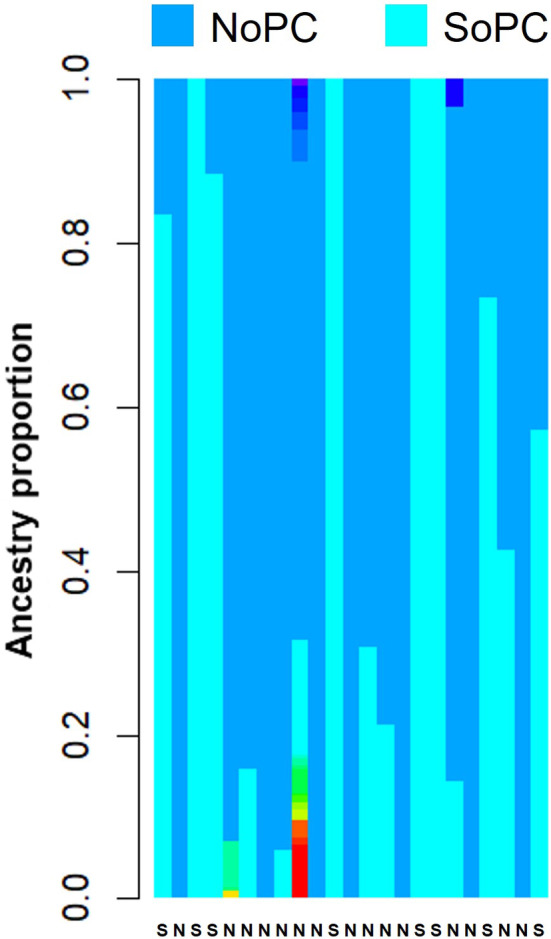
Closer detail to highlight the TRNP results from the ADMIXTURE *K* = 28 model in Figure A4. The TRNP horses are split into two exclusive clusters that correspond with their geographical region (North or South of Paddock Creek) within the park.

At *K* = 28, the TRNP horses were split into two clusters (Figure A4). The division was aligned with the geographical categories assigned to TRNP individuals for population structure analysis. Again, no individual from any other breed had more than a trace assignment to either TRNP cluster, and only three TRNP individuals had genome proportions between 0.0002 and 0.056 assigned to any other clusters (Figure A5).

### 3.4 Estimates of inbreeding

TRNP horses had relatively high values for inbreeding coefficients compared to other breeds. The mean *f* of TRNP horses was 0.180, while the mean *f* for all other breeds in the dataset was 0.116 (standard deviation of 0.079). Only seven breeds had a mean *f* higher than the TRNP horses (Tables A4 and A5).

**FIGURE 5:**
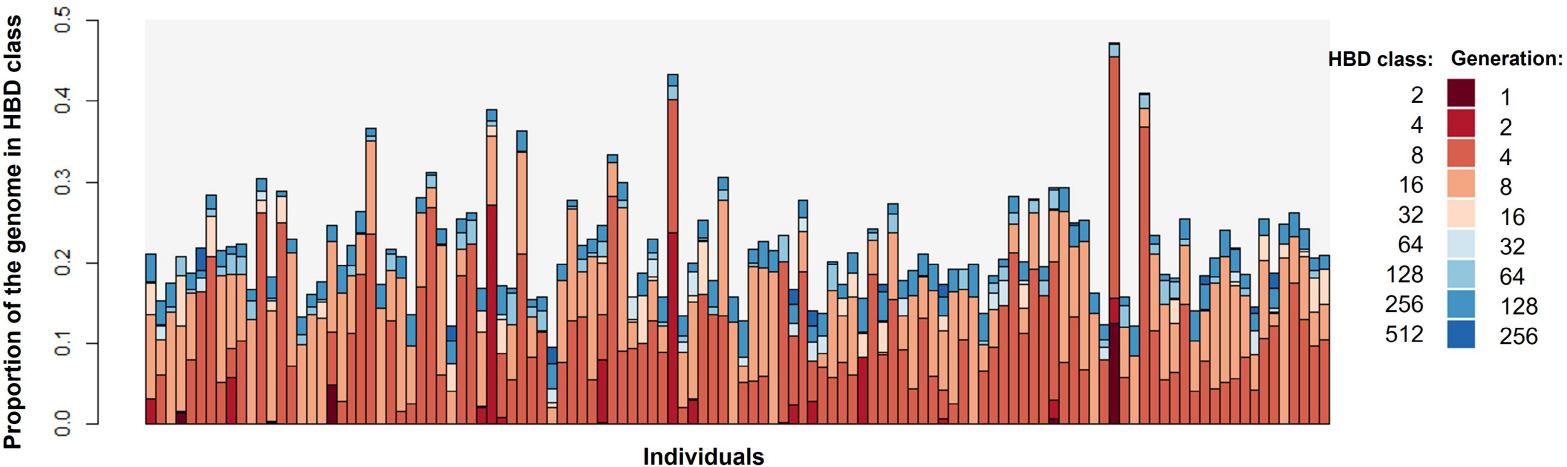
Proportion of the genome in each homozygosity by descent (HBD) class for all 118 TRNP individuals as estimated by RZooRoH. HBD classes represent inbreeding level based on number of generations removed to common ancestor, where HBD class 2 corresponds with 1 generation to common ancestor, HBD class 4 with 2 generations, etc.

**FIGURE 6:**
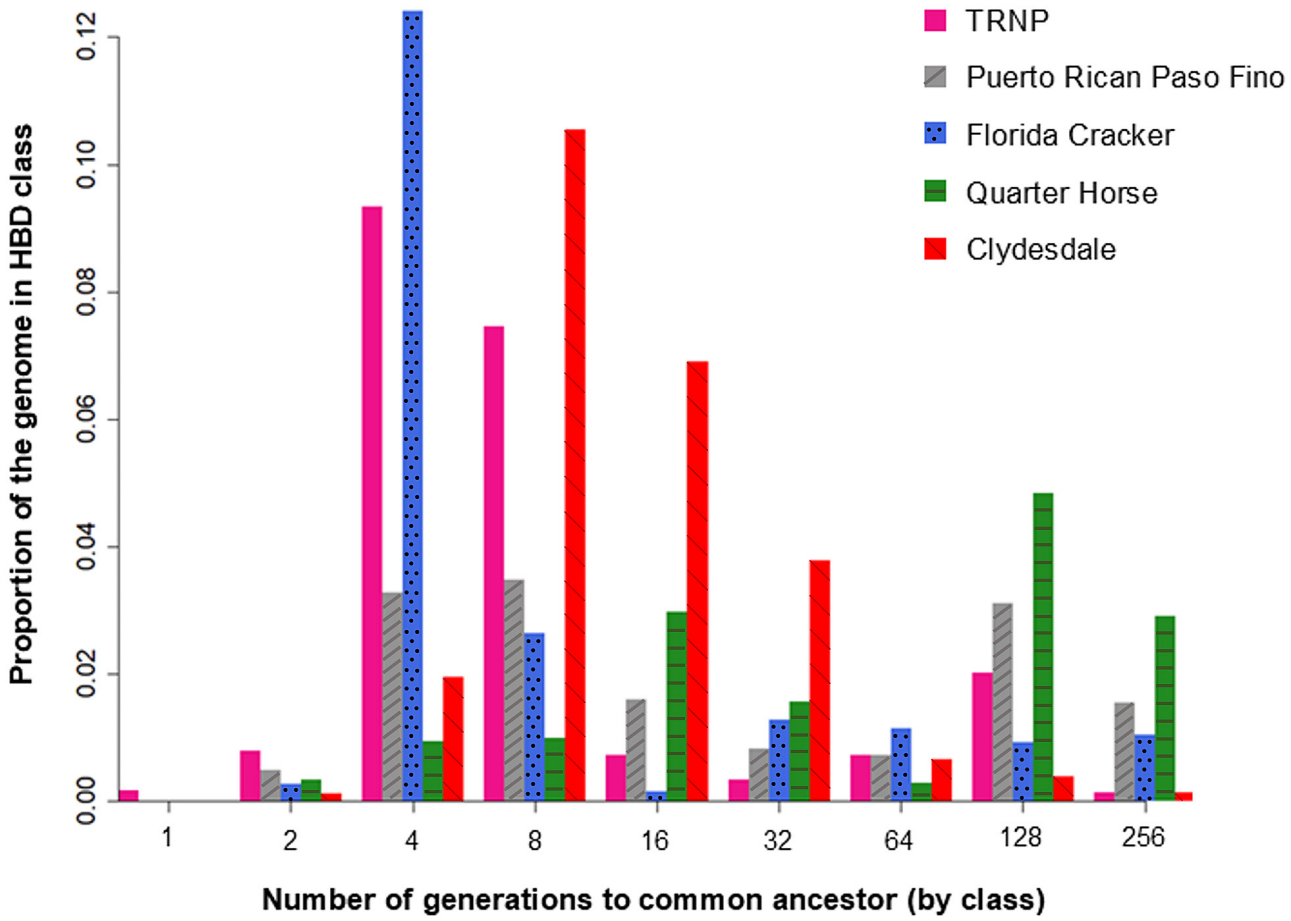
Average proportion of the genome in each generation class for all 118 TRNP horses in comparison to four other breeds of known population history, as estimated by RZooRoH. Clydesdale and Florida Cracker have undergone recent bottlenecks, Puerto Rican Paso Fino an older bottleneck, and Quarter Horses have been recently admixed. HBD classes represent inbreeding level based on number of generations removed to common ancestor, with lower generation numbers corresponding to more recent inbreeding. TRNP horses have highest presence of HBD segments in classes 4 and 8 generations ago, which corresponds with the initial population bottleneck from the time of the park's establishment.

RZooRoH identified 9195 HBD sequences among the 118 TRNP samples, on all autosomes and in all individuals. The mean proportion of the genome covered by HBD segments for TRNP individuals was 0.22 (SD 0.066; range 0.095–0.47; median 0.21; IQR 0.17–0.25) (Figure 5). The highest proportions were in generation classes 4 and 8 (Figures 5 and 6). With a generation time of 10 years this corresponds to common ancestors approximately 40–80 years ago, suggesting bottlenecking or founder events around that timeframe. For comparison, Figure 6 also shows the Clydesdale and Florida Cracker, which have both undergone recent genetic bottlenecks, the Puerto Rican Paso Fino, which experienced a more distant bottleneck during the importation of Spanish horses to the Americas, and the Quarter Horse, which has multiple sources of recent admixture.

### 3.5 TRNP population structure

**FIGURE 7:**
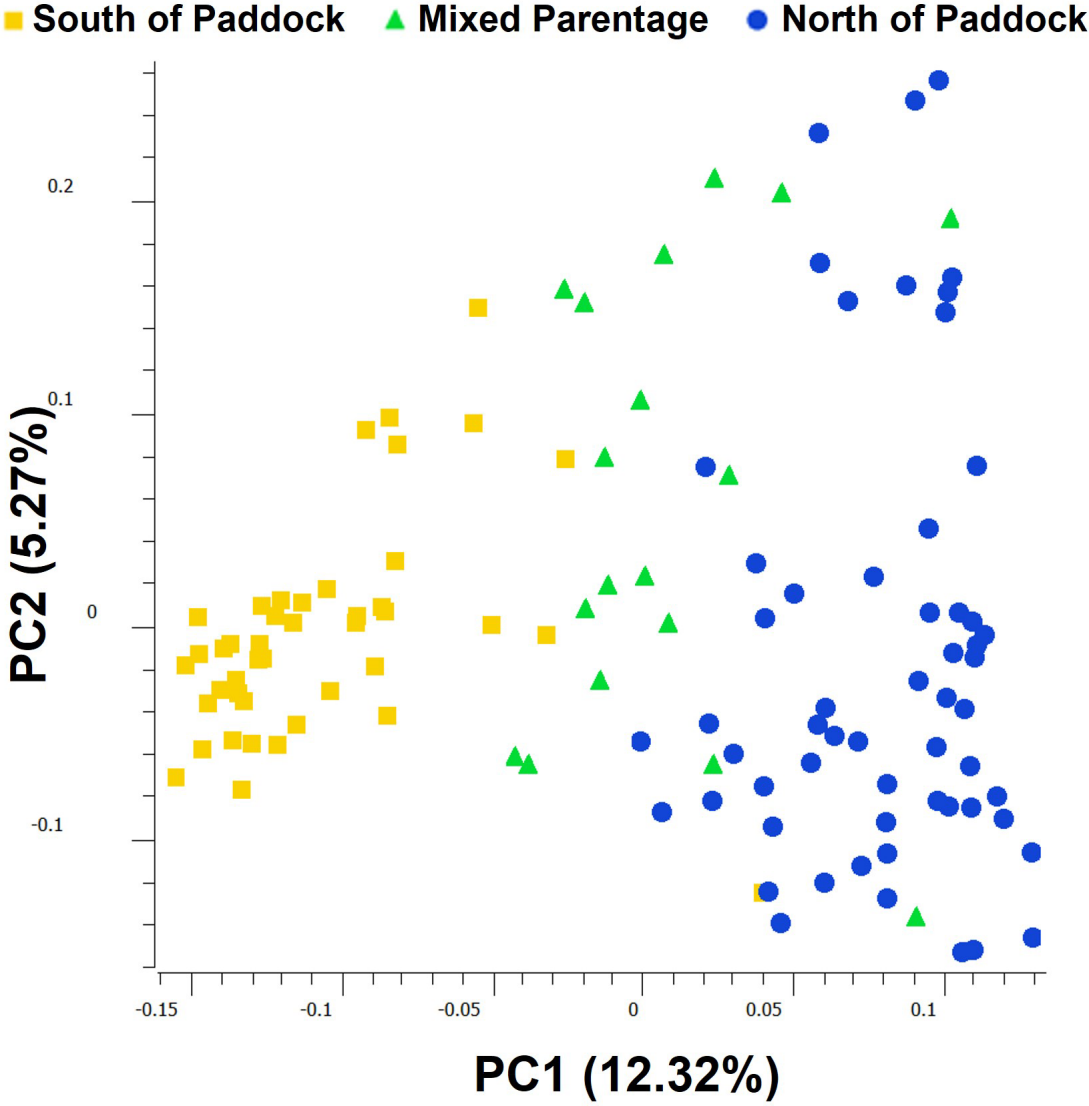
Principal components analysis of all 118 TRNP samples, with individuals labeled by geographic region within the South Unit of TRNP. The first two principal components explain 12.32% and 5.27% of the genetic variation, respectively. Individuals are generally sorted by geographic category along PC1.

On the plot of PC1 by PC2 for the 118 TRNP samples, individuals are noticeably sorted by geographic category along PC1 (Figure 7). PC1 captures 12.32% of the genetic variation in the dataset, and PC2 captures 5.27%. The individuals located South of Paddock Creek (SoPC) are more closely grouped than are individuals North of Paddock Creek (NoPC). Individuals of mixed parentage are between and among the two categories. On PC2, the NoPC points have a wider spread than do the SoPC points.

## 4 DISCUSSION

By using multiple approaches to analyze the population genetics of TRNP horses, we identified overall patterns that reflect the history of this herd. These analyses place the TRNP horses well within the diversity seen in modern domestic horses, but do not show a strong signal of relatedness to any one breed, consistent with previous work and the evolutionarily recent development of most horse breeds. Based on park records, it is known that the TRNP herd has had genetic influxes from multiple sources. Admixture between breeds and the recent isolation of horse breeds makes it challenging to determine ancestry in the more distant past. As these breeds have experienced continued artificial selection for certain characteristics, they differ from horse populations that existed in the late 1800s. Admixture can create new allele combinations and frequencies, contributing to the apparent population differentiation. However, some patterns emerge across these analyses.

The PCA plot reflects variation in genotypes and identifies unique populations and associations with phenotypic traits for the sampled breeds. Highly specialized breeds can be found as distinct from other breeds due to strong selection pressures and inbreeding, and the plot can also show evidence of admixture between populations (Petersen, Mickelson, Cothran, et al., 2013). The TRNP points are not as tightly clustered together as some of the other breeds, indicating admixture between multiple sources in their recent history (McVean, 2009). Some TRNP points are separated from the center of the cluster, falling toward the Shires and other draft breeds. This separation may reflect the influence of the Shire‐Paint stallion introduced to the park in the 1980s. On the PCA plot, the five Spanish‐type breeds are all tightly clustered together. The TRNP points align with Spanish‐type breeds on PC1 as well as with such breeds as Franches‐Montagnes, Morgan, and Tuva, but are separated from them on PC2, potentially indicating some affinity between the herd and these breeds.

The general distribution of domestic breeds in PCA is consistent across multiple reports (Funk et al., 2020; Ovchinnikov et al., 2018). Though direct comparisons cannot be made across separate analyses, there are some interesting similarities between our results and the results of another study of two feral Canadian populations evaluated with the same Petersen, Mickelson, Rendahl, et al. (2013) dataset. A large population of feral horses (“Alberta Foothills”) that has ranged in size from 1000 to 1700 individuals and likely experienced continual gene inflow from multiple draft breeds and Quarter Horses/Paints appeared in a generally similar position to the TRNP horses in a PCA plot (Tollett, 2018). A second, isolated feral population of about 500 individuals (“Sable Island”) was more tightly clustered on PC1 and PC2 but was noticeably separated from the main cloud of points on PC3 (Tollett, 2018). The TRNP herd, though considerably smaller than the Sable Island herd, does not show such divergence on PC3.

The maximum likelihood tree reflects the pattern seen in the PCA plot, with Thoroughbreds located in one portion of the topology while draft horses and ponies are found on the other side of the tree. The TRNP horses are found to be more similar to draft breeds and particularly the Shires, Clydesdales, and Fell Ponies, supporting the idea that the herd retains influence from the introduced Shire‐Paint stallion. If this influence does come substantially from that individual, the more recent timeframe of his introduction in comparison to the development of most breeds would contribute to the lower value of bootstrap confidence observed. Due to the local popularity of Percheron horses in the late 1800s, though, the genetic contribution of draft breeds may also have been present before his introduction (Crawford, 1931; Huidekoper, 1947; McLaughlin, 1989). However, there is support for separation of the Spanish breeds from the TRNP horses, suggesting that these Spanish breeds have had limited genetic influence on TRNP horses in recent history compared with draft breeds.

During domestication, artificial selection for specialized traits, along with transport and husbandry of horses, resulted in breeds or types within a short evolutionary timeframe. Our analyses indicate that horse breed relationships are reconstructed in a star‐like phylogeny, with short internal branches and long external branches, indicating rapid rates of diversification due to strong selection. Because horse breeds successfully interbreed, it is difficult to reconstruct that genetic history with a simple bifurcating phylogeny. This difficulty is reflected in the low nodal support for internal nodes. Recombination, or gene flow between branches, can affect the shape of phylogenetic trees, lengthening the terminal branches (Li et al., 2019; Schierup & Hein, 2000). This pattern is commonly seen within domestic species, such as dogs, cattle and water buffalo, goats, and chickens (Mannen et al., 2020; Quan et al., 2020; Rout et al., 2008; Sun et al., 2020; Vonholdt et al., 2010). Other trees constructed with different data show a similar phylogenetic pattern for horse breeds (Felkel et al., 2018; Khanshour et al., 2013; Vilà et al., 2001).

Based on the oral history of the herd, we would expect to see some genetic similarity between the TRNP horses and one or all of the Spanish breeds. In the PCA, the TRNP horses are close to Spanish breeds on PC1 but diverge from those breeds on PC2. Relatively high *F*_ST_ values indicate that there is little recent Spanish contribution to the herd. Further, the maximum likelihood tree and the ADMIXTURE analysis separate the Spanish breeds from the TRNP horses. Based on these observations, it seems that the present‐day herd is not closely related to the Spanish breeds. Considering the history of horses in the Americas, though, we cannot rule out previous Spanish influences. However, there were approximately 70 years of few records between the purported import of Spanish type horses into the local area in the 1880s and the 1950s when herd management began, during which local ranchers were known to cross “Indian type” horses with European breeds (McLaughlin, 1989). Thus, any Spanish lineage would most likely have experienced admixture before the founding of the park. Additionally, most horses in the park in the 1950s were branded, suggesting considerable influence of 20th century ranching practices on herd composition.

A common thread across the ADMIXTURE, phylogeny, and PCA results is that TRNP horses are a distinct population in comparison to these domestic horse breeds. Genetic differentiation can be driven by selection, mutation, reduced gene flow, genetic drift, and nonrandom mating. Although the oldest formal breed registries have only existed for approximately 200 years, horses have been under artificial selection during their domestication for at least 4000 years (Orlando, 2020). The TRNP herd has not experienced artificial selection for specific characteristics but has had limited gene flow and a small population size (80–200 individuals) since the 1950s. A few individuals have been introduced over the history of the herd; however, the last intentional introduction as part of a management decision occurred in the 1980s. Genetic differentiation likely resulted from the isolation and repeated bottleneck events experienced by the TRNP herd, resulting in genetic drift. Essentially, the TRNP horses are more similar to each other in allelic combinations than they are to any other horse breeds.

Ovchinnikov et al. (2018) reported low values of genetic variability (observed heterozygosity and allelic diversity) in the TRNP herd compared to both domestic breeds and other feral herds. This is also reflected in inbreeding coefficients from genome‐wide analysis of SNPs, with the TRNP horses having higher values of *f* than most other breeds. The *F*_ST_ values between TRNP and other breeds were all near or higher than the average among all breeds. *F*_ST_ measures genetic differentiation between populations using allele frequencies and can indicate reduced gene flow between those populations. While the TRNP horses are placed among the draft breeds on the phylogenetic tree, the Shire and Clydesdale also have some of the highest values for inbreeding coefficient, *f*. In combination with the high mean *f* of the TRNP population, this resulted in high values of *F*_ST_ between these breeds, despite some shared ancestry. We know that the TRNP population has been isolated for many years, and the lack of recent gene flow with other breeds is supported by these high *F*_ST_ values.

Though *F*_ST_ values are lower between TRNP and Paint and Quarter Horses than TRNP and any draft breed, other results do not single out these breeds as recent contributors to the park population. Morgan horses are another putative source of TRNP ancestry based on PCA results; Morgan horses are the oldest remaining North American horse breed, originating in the late 1700s and early 1800s for use on farms (Battell, 1894). Perhaps the ranch horses of early western North Dakota were genetically similar to the early work horses of the eastern US and to the early Quarter Horses of Texas, before their differentiation into strictly kept breeds. It is possible that these early work horses contributed to the TRNP population.

The ROH analysis also shows that the TRNP horses have experienced relatively recent inbreeding. The high proportion of HBD segments in the genome due to inheritance from common ancestors four and eight generations ago coincides with the herd's isolation and known bottleneck events. The initial bottleneck occurred 60–70 years ago (6–7 generations) in the 1950s and 60s following the establishment of the park, when the majority of the horses on the land were rounded up and returned to their owners at the same time as the park perimeter was fenced and the remaining population was isolated. A reduction of the TRNP population to 16 individuals, followed by low gene flow into the population, explains the current presence of large chromosome segments inherited from common ancestors. The presence of these HBD segments and a relatively high inbreeding coefficient suggest that the bottlenecks experienced in the recent history of the TRNP herd have affected genetic diversity of the population. In the case of the individual horse with the highest presence of HBD segments (0.47), pedigree records indicate that this individual likely had the same stallion‐mare mating in the P2 generation on one side and the P3 generation on the other. Chromosome 1 for this individual appeared almost entirely homozygous.

However, HBD segments are likely overestimated in our dataset, due to the density of SNPs called in the array. Lavanchy and Goudet (2023) demonstrated that SNP density is an important metric in accurately assessing HBD segment presence and recommend using high‐density (>11 SNPs/Mb) datasets. While more recent inbreeding and longer HBD segments are easier to estimate, lower SNP density can result in overestimation of small segments. Still, since the TRNP population is small and inbred, some of the unsampled portions of the genome are likely to also be homozygous.

RZooRoH produced HBD class results consistent with the known history of several other breeds, indicating that the TRNP results for HBD classes were likely reasonable. For example, Clydesdales experienced a bottleneck following agricultural mechanization and their use in WWI and WWII during the 1920s–1940s (Hendricks, 1995). RZooRoH assigned a higher proportion of their genome to 8 generations ago, consistent with this timeframe. A more recent and severe reduction in population size occurred with the Florida Cracker with only 31 individuals present in 1989 (Florida Cracker Horse Association; Conant et al., 2012), which is reflected by the highest HBD level occurring in the four generation class. These breeds and the TRNP horses have a clear signature of inbreeding compared with the Quarter Horse, a breed with very large population size and admixture from multiple sources, but which has only formally existed since 1940. In fact most horse breeds and breed registries were only established within the last few centuries, alongside an increased awareness of heritability (Hendricks, 1995). The HBD classes here extend out to hundreds of generations and thousands of years ago and suggest a level of inbreeding that may be present for the species in general which occurred around domestication events.

As we hypothesized, large scale roundup actions have likely caused genetic bottlenecks in the herd and led to inbreeding, with the initial reduction of herd size at the time of the installation of the perimeter fence likely producing the largest effect. Inbreeding can also be exacerbated by subpopulation differentiation within a population. Smaller subpopulations can experience increased effects of genetic drift and an overall loss of heterozygosity within the population as a whole. Within the TRNP herd there is evidence of some population structure, as seen in the TRNP PCA and the splitting of the TRNP ADMIXTURE cluster at *K* = 28 (Figure A5). Observational data shows that many of the TRNP horses have a behavioral tendency to inhabit a regular area within the park and disperse to join other family bands that also frequent the same area. Though the Paddock Creek corridor is not a restrictive physical barrier, there seems to be enough site fidelity to contribute to nonrandom mating and the development of genetic population structure. However, there is still frequent gene flow within the population, as evidenced by the mixed parentage individuals, and likely enough gene flow to prevent substantial subpopulation differentiation.

## 5 CONCLUSIONS

The use of much larger numbers of markers in a SNP array provided a more in‐depth evaluation of this feral population than had previously been possible with limited markers. While the TRNP population continued to group separately from other breeds, consistent with previous work, we did detect genetic relationships where previous work had been inconclusive. The strongest observed similarity is between TRNP horses and some draft breeds, based on phylogeny and ADMIXTURE relationships. In particular, the placement of the TRNP horses on a branch next to Shires, Clydesdales, and Fell Ponies in the maximum likelihood tree indicates that descendants of the introduced Shire‐Paint stallion persist in the present‐day herd. The inbreeding analyses indicate that the TRNP herd has experienced inbreeding and differentiation from other breeds, likely due to genetic drift, bottleneck events, and limited gene flow. Historical management actions likely exacerbated the inbreeding levels within the population, especially the original population bottleneck and initial removal attempt at the time of the perimeter fence installation in the 1950s.

## 6 IMPLICATIONS

If a reproductive herd is to be maintained, approaches to reduce the effect of continued isolation on genetic diversity can be considered. Genetic diversity in a closed population may be increased with introduction of new individuals. Periodic introductions could be used to counter the effects of genetic drift. Depending on long term management objectives, individuals for introduction could be chosen from a more genetically distant population to maximize variation, or from a population with a more similar background. Analysis of additional samples from other feral herds across the country might identify more relationships, potentially suggesting another similar source from which to select animals for introduction.

## 7 PERMIT INFORMATION

2020 biopsy samples were collected under research permit number THRO‐2020‐SCI‐0013 and NPS IACUC approval (ND_THRO_McCann_HorseBiopsyDarting_2020.A1).

## AUTHOR CONTRIBUTIONS

**Blake E. McCann:** Conceptualization (equal); writing – review and editing (equal). **Melissa A. Thompson:** Formal analysis (lead); writing – original draft (lead); writing – review and editing (equal). **Rebecca B. Simmons:** Supervision (equal); writing – review and editing (equal). **Turk Rhen:** Writing – review and editing (equal).

## CONFLICT OF INTEREST STATEMENT

The authors declare no conflict of interest.

## Data Availability

Genotype data used in this study were submitted to the European Variation Archive (EVA) under accession number PRJEB64774 and will be openly available at https://www.ebi.ac.uk/eva/?eva‐study=PRJEB64774 following a 1 year embargo.

## Appendix

**TABLE A1:** Number of individuals of each domestic breed included in analyses.

Breed	# Of individuals
Akhal‐Teke	17
Andalusian	18
Arabian	24
Belgian	30
Clydesdale	24
Caspian	17
Exmoor	24
Fell Pony	21
Finnhorse	27
Florida Cracker	7
Franches‐Montagnes	19
French Trotter	17
Hanoverian	15
Icelandic	25
Lusitano	24
Maremmano	24
Miniature	21
Mangalarga Paulista	15
Mongolian	19
Morgan	40
New Forest Pony	15
Norwegian Fjord	21
North Swedish Horse	19
Percheron	23
Peruvian Paso	21
Puerto Rican Paso Fino	20
Paint	25
Quarter Horse	40
Saddlebred	25
Shetland	27
Shire	23
Standardbred	40
Swiss Warmblood	14
Thoroughbred	36
Tuva	15

**TABLE A2:** First ten principal components from PCA.

Principal component	Percentage of variance explained/eigenvalues
1	36.90
2	14.06
3	11.23
4	10.16
5	8.98
6	8.36
7	7.42
8	7.18
9	6.07
10	5.63

**TABLE A3:** Pairwise *F*_ST_ values calculated for 36 populations/breeds.

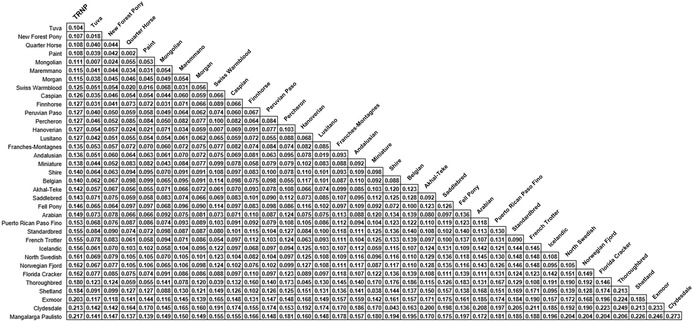

*Note*: Rows are sorted by value in comparison to TRNP.

**TABLE A4:** Mean inbreeding coefficient *f* by breed, with standard deviation, minimum and maximum values.

Breed	Mean *f*	SD	MIN	MAX
Hanoverian	−0.007	0.016	−0.041	0.024
Swiss Warmblood	−0.003	0.025	−0.040	0.064
Paint	0.001	0.023	−0.038	0.061
Quarter Horse	0.005	0.031	−0.039	0.110
Maremmano	0.005	0.022	−0.033	0.044
Thoroughbred	0.046	0.027	−0.021	0.102
Caspian	0.062	0.043	−0.010	0.148
New Forest Pony	0.066	0.024	0.025	0.112
French Trotter	0.068	0.028	0.018	0.127
Mongolian	0.070	0.037	−0.078	0.099
Tuva	0.071	0.044	0.016	0.165
Saddlebred	0.082	0.024	0.032	0.129
Akhal‐Teke	0.082	0.038	0.019	0.130
Peruvian Paso	0.088	0.034	0.050	0.149
Morgan	0.096	0.074	0.014	0.302
Lusitano	0.099	0.053	0.013	0.194
Finnhorse	0.106	0.018	0.076	0.166
Franches‐Montagnes	0.109	0.047	0.041	0.233
Arabian	0.109	0.060	0.039	0.321
Standardbred	0.117	0.038	0.047	0.206
Andalusian	0.129	0.067	0.050	0.320
Miniature	0.132	0.024	0.096	0.210
Puerto Rican Paso Fino	0.141	0.061	0.035	0.324
Percheron	0.144	0.025	0.105	0.187
Fell Pony	0.155	0.028	0.121	0.228
Icelandic	0.157	0.032	0.125	0.281
Belgian	0.157	0.026	0.098	0.201
Florida Cracker	0.159	0.136	0.003	0.365
TRNP	0.180	0.094	0.018	0.466
Norwegian Fjord	0.184	0.031	0.130	0.257
North Swedish	0.187	0.038	0.120	0.266
Shire	0.194	0.088	−0.040	0.298
Shetland	0.237	0.056	0.158	0.408
Mangalarga Paulista	0.252	0.049	0.170	0.351
Exmoor	0.285	0.086	0.104	0.568
Clydesdale	0.310	0.046	0.189	0.374

**TABLE A5:** Values of inbreeding coefficient *f* for all 118 TRNP samples, as well as the number of observed homozygous loci and the number of expected homozygous loci as calculated by SVS.

*f*	# Markers	# Observed homozygotes	# Expected homozygotes	*f*	# Markers	# Observed homozygotes	# Expected homozygotes
−0.166	45,793	26,603	29329.30	−0.022	45,785	28,969	29326.44
−0.149	45,685	26,813	29259.76	−0.020	45,761	28,985	29312.11
−0.146	43,455	25,715	27969.38	−0.017	45,794	29,044	29331.60
−0.142	45,796	26,997	29332.39	−0.017	45,803	29,055	29336.75
−0.127	45,803	27,244	29336.49	−0.010	45,785	29,167	29325.38
−0.125	45,795	27,282	29332.16	−0.005	45,790	29,250	29328.92
−0.121	45,766	27,320	29315.20	−0.005	45,796	29,255	29331.91
−0.114	45,785	27,449	29326.62	−0.004	45,785	29,255	29325.62
−0.113	45,795	27,464	29330.87	−0.004	45,796	29,266	29331.33
−0.109	45,603	27,423	29211.23	0.002	45,802	29,362	29336.70
−0.108	45,804	27,562	29337.65	0.002	45,756	29,344	29306.15
−0.107	45,553	27,422	29179.40	0.003	45,774	29,367	29317.91
−0.106	45,790	27,580	29328.29	0.003	45,805	29,394	29338.23
−0.104	45,807	27,622	29339.30	0.003	45,795	29,388	29331.90
−0.103	45,803	27,633	29336.76	0.004	45,788	29,389	29326.76
−0.103	45,801	27,633	29334.89	0.006	42,728	27,637	27549.09
−0.102	45,796	27,648	29332.50	0.006	45,793	29,434	29330.63
−0.099	45,415	27,475	29087.60	0.011	45,709	29,462	29277.63
−0.098	45,791	27,720	29329.38	0.013	45,797	29,550	29333.38
−0.092	45,793	27,821	29330.03	0.018	45,788	29,627	29327.66
−0.090	45,377	27,588	29055.69	0.022	45,795	29,696	29331.84
−0.088	45,804	27,894	29337.46	0.022	45,790	29,692	29327.72
−0.086	45,735	27,885	29294.44	0.024	45,796	29,723	29331.87
−0.077	45,775	28,047	29318.10	0.027	45,783	29,768	29323.68
−0.074	45,791	28,106	29329.28	0.030	45,726	29,790	29290.24
−0.069	45,556	28,051	29176.05	0.031	45,703	29,783	29270.62
−0.066	45,709	28,194	29276.72	0.033	45,804	29,886	29337.59
−0.065	45,776	28,257	29319.24	0.034	45,801	29,902	29335.45
−0.064	45,779	28,264	29321.15	0.036	45,763	29,903	29311.07
−0.063	45,798	28,302	29333.37	0.036	45,797	29,928	29333.48
−0.062	45,805	28,312	29338.16	0.040	45,786	29,984	29325.14
−0.062	45,794	28,307	29331.18	0.040	45,786	29,992	29327.34
−0.062	45,750	28,284	29302.06	0.042	45,802	30,033	29336.43
−0.060	45,685	28,271	29260.95	0.047	45,802	30,116	29336.88
−0.059	45,743	28,326	29299.18	0.047	45,777	30,101	29320.66
−0.058	45,798	28,385	29334.00	0.066	45,787	30,415	29326.84
−0.057	45,790	28,398	29328.82	0.067	45,798	30,441	29333.57
−0.056	45,796	28,412	29330.90	0.069	45,769	30,447	29315.20
−0.055	45,803	28,438	29337.12	0.069	45,752	30,445	29305.89
−0.053	45,798	28,457	29334.12	0.071	45,792	30,498	29329.68
−0.053	45,801	28,471	29335.68	0.072	45,767	30,490	29312.09
−0.052	43,485	27,019	27835.87	0.072	45,808	30,522	29339.77
−0.049	45,791	28,525	29328.52	0.079	45,802	30,631	29336.04
−0.041	45,765	28,630	29311.84	0.081	45,800	30,668	29334.73
−0.041	45,802	28,665	29336.72	0.083	45,743	30,672	29300.52
−0.041	45,720	28,615	29284.88	0.088	45,791	30,774	29328.69
−0.040	45,741	28,643	29299.15	0.093	45,760	30,847	29309.74
−0.039	45,780	28,688	29323.74	0.100	45,774	30,959	29319.53
−0.036	45,805	28,744	29338.28	0.103	45,798	31,028	29334.24
−0.036	45,804	28,747	29337.14	0.117	45,686	31,177	29262.51
−0.035	45,799	28,755	29334.62	0.123	45,793	31,348	29330.17
−0.034	45,774	28,762	29317.90	0.134	45,742	31,508	29298.07
−0.029	45,802	28,858	29336.19	0.142	45,795	31,663	29331.35
−0.027	45,792	28,890	29328.37	0.186	45,796	32,394	29332.55
−0.026	45,692	28,844	29268.98	0.200	45,792	32,615	29330.60
−0.025	45,312	28,634	29048.60	0.225	45,778	33,026	29320.35
−0.024	45,787	28,928	29327.03	0.232	45,758	33,130	29309.27
−0.024	45,498	28,778	29162.53	0.268	45,785	33,741	29324.82
−0.022	45,787	28,958	29327.23	0.324	45,772	34,642	29317.30

## References

[ece311197-bib-0001] Alexander, D. H. , Novembre, J. , & Lange, K. (2009). Fast model‐based estimation of ancestry in unrelated individuals. Genome Research, 19, 1655–1664. 10.1101/GR.094052.109 19648217 PMC2752134

[ece311197-bib-0002] Battell, J. (1894). The Morgan horse and register. Register Printing Company. Register Printing Company.

[ece311197-bib-0003] Bertrand, A. R. , Kadri, N. K. , Flori, L. , Gautier, M. , & Druet, T. (2019). RZooRoH: An R package to characterize individual genomic autozygosity and identify homozygous‐by‐descent segments. Methods in Ecology and Evolution, 10, 860–866.

[ece311197-bib-0004] Ceballos, F. C. , Joshi, P. K. , Clark, D. W. , Ramsay, M. , & Wilson, J. F. (2018). Runs of homozygosity: Windows into population history and trait architecture. Nature Reviews Genetics, 19, 220–234. 10.1038/nrg.2017.109 29335644

[ece311197-bib-0005] Conant, E. K. , Juras, R. , & Cothran, E. G. (2012). A microsatellite analysis of five colonial Spanish horse populations of the southeastern United States. Animal Genetics, 43, 53–62. 10.1111/J.1365-2052.2011.02210.X 22221025

[ece311197-bib-0044] Cosgrove, E. J. , Sadeghi, R. , Schlamp, F. , Holl, H. M. , Moradi‐Shahrbabak, M. , Miraei‐Ashtiani, S. R. , Abdalla, S. , Shykind, B. , Troedsson, M. , Stefaniuk‐Szmukier, M. , Prabhu, A. , Bucca, S. , Bugno‐Poniewierska, M. , Wallner, B. , Malek, J. , Miller, D. C. , Clark, A. G. , Antczak, D. F. , & Brooks, S. A. (2020). Genome diversity and the origin of the Arabian horse. Scientific Reports, 10(1). 10.1038/s41598-020-66232-1 PMC7298027 32546689

[ece311197-bib-0006] Cothran, E. G. (1992). Genetic marker analysis of the Theodore Roosevelt, National Park Feral Horse Herd. National Park Service.

[ece311197-bib-0007] Cothran, E. G. (2000). Analysis of genetic variation in the feral horse herd of the Theodore Roosevelt National Park in 2000 . National Park Service.

[ece311197-bib-0008] Crawford, L. F. (1931). History of North Dakota. American Historical Society.

[ece311197-bib-0009] Felkel, S. , Vogl, C. , Rigler, D. , Jagannathan, V. , Leeb, T. , Fries, R. , Neuditschko, M. , Rieder, S. , Velie, B. , Lindgren, G. , Rubin, C. J. , Schlötterer, C. , Rattei, T. , Brem, G. , & Wallner, B. (2018). Asian horses deepen the MSY phylogeny. Animal Genetics, 49, 90–93. 10.1111/age.12635 29333704

[ece311197-bib-0010] Florida Cracker Horse Association [WWW Document] . (2022). https://floridacrackerhorseassociation.com/about‐us/

[ece311197-bib-0011] Funk, S. M. , Guedaoura, S. , Juras, R. , Raziq, A. , Landolsi, F. , Luís, C. , Martínez, A. M. , Musa Mayaki, A. , Mujica, F. , Oom, M. d. M. , Ouragh, L. , Stranger, Y. M. , Vega‐Pla, J. L. , & Cothran, E. G. (2020). Major inconsistencies of inferred population genetic structure estimated in a large set of domestic horse breeds using microsatellites. Ecology and Evolution, 10, 4261–4279. 10.1002/ECE3.6195 32489595 PMC7246218

[ece311197-bib-0045] Gurgul, A. , Jasielczuk, I. , Semik‐Gurgul, E. , Pawlina‐Tyszko, K. , Stefaniuk‐Szmukier, M. , Szmatoła, T. , Polak, G. , Tomczyk‐Wrona, I. , & Bugno‐Poniewierska, M. (2019). A genome‐wide scan for diversifying selection signatures in selected horse breeds. PLoS One, 14(1), e0210751. 10.1371/journal.pone.0210751 30699152 PMC6353161

[ece311197-bib-0012] Harmon, D. (1986). At the open margin: The NPS's administration of Theodore Roosevelt National Park. Theodore Roosevelt Nature and History Association.

[ece311197-bib-0013] Hendricks, B. L. (1995). International encyclopedia of horse breeds. University of Oklahoma Press.

[ece311197-bib-0014] Huidekoper, A. C. (1947). My experience and investment in the bad lands of Dakota and some of the men I met there. Wirth Brothers.

[ece311197-bib-0015] Khanshour, A. , Conant, E. , Juras, R. , & Cothran, E. G. (2013). Microsatellite analysis of genetic diversity and population structure of Arabian horse populations. The Journal of Heredity, 104, 386–398. 10.1093/JHERED/EST003 23450090

[ece311197-bib-0016] Lacy, R. C. , & Pollak, J. P. (2021). Vortex: A stochastic simulation of the extinction process . Chicago Zoological Society.

[ece311197-bib-0017] Lavanchy, E. , & Goudet, J. (2023). Effect of reduced genomic representation on using runs of homozygosity for inbreeding characterization. Molecular Ecology Resources, 23, 787–802. 10.1111/1755-0998.13755 36626297

[ece311197-bib-0018] Lewis, P. O. (2003). NCL: A C++ class library for interpreting data files in NEXUS format. Bioinformatics, 19, 2330–2331. 10.1093/BIOINFORMATICS/BTG319 14630669

[ece311197-bib-0019] Li, G. , Figueiro, H. V. , Eizirik, E. , & Murphy, W. J. (2019). Recombination‐aware phylogenomics reveals the structured genomic landscape of hybridizing cat species. Molecular Biology and Evolution, 36, 2111–2126. 10.1093/molbev/msz139 31198971 PMC6759079

[ece311197-bib-0020] Mannen, H. , Yonezawa, T. , Murata, K. , Noda, A. , Kawaguchi, F. , Sasazaki, S. , Olivieri, A. , Achilli, A. , & Torroni, A. (2020). Cattle mitogenome variation reveals a post‐glacial expansion of haplogroup P and an early incorporation into northeast Asian domestic herds. Scientific Reports, 10, 20842. 10.1038/s41598-020-78040-8 33257722 PMC7704668

[ece311197-bib-0021] Marlow, C. B. , Gagnon, L. C. , Irby, L. R. , & Raven, M. R. (1992). Feral horse distribution, habitat use and population dynamics in Theodore Roosevelt National Park. National Park Service.

[ece311197-bib-0022] McLaughlin, C. (1989). The history and status of the wild horses of Theodore Roosevelt National Park . Theodore Roosevelt Nature and History Association.

[ece311197-bib-0023] McVean, G. (2009). A genealogical interpretation of principal components analysis. PLoS Genetics, 5, e1000686. 10.1371/JOURNAL.PGEN.1000686 19834557 PMC2757795

[ece311197-bib-0024] Miller, M. A. , Pfeiffer, W. , & Schwartz, T. (2010). Creating the CIPRES Science Gateway for inference of large phylogenetic trees. 2010 Gateway Computing Environments Workshop (GCE). 10.1109/gce.2010.5676129

[ece311197-bib-0025] National Park Service . (1978). Environmental assessment: Proposed feral horse reduction. Theodore Roosevelt National Memorial Park.

[ece311197-bib-0026] National Research Council . (2013). Using science to improve the BLM wild horse and burro program: A way forward. National Research Council.

[ece311197-bib-0027] Orlando, L. (2020). Ancient genomes reveal unexpected horse domestication and management dynamics. BioEssays, 42, e1900164. 10.1002/bies.201900164 31808562

[ece311197-bib-0028] Ortiz, E. M. (2019). vcf2phylip v2.0: Convert a VCF matrix into several matrix formats for phylogenetic analysis . 10.5281/ZENODO.2540861

[ece311197-bib-0029] Ovchinnikov, I. V. , Dahms, T. , Herauf, B. , McCann, B. , Juras, R. , Castaneda, C. , & Cothran, E. G. (2018). Genetic diversity and origin of the feral horses in Theodore Roosevelt National Park. PLoS One, 13, e0200795. 10.1371/journal.pone.0200795 30067807 PMC6070244

[ece311197-bib-0030] Peripolli, E. , Munari, D. P. , Silva, M. V. G. B. , Lima, A. L. F. , Irgang, R. , & Baldi, F. (2016). Runs of homozygosity: Current knowledge and applications in livestock. Animal Genetics, 48, 255–271. 10.1111/age.12526 27910110

[ece311197-bib-0031] Petersen, J. L. , Mickelson, J. R. , Cothran, E. G. , Andersson, L. S. , Axelsson, J. , Bailey, E. , Bannasch, D. , Binns, M. M. , Borges, A. S. , Brama, P. , da Câmara Machado, A. , Distl, O. , Felicetti, M. , Fox‐Clipsham, L. , Graves, K. T. , Guérin, G. , Haase, B. , Hasegawa, T. , Hemmann, K. , … McCue, M. E. (2013). Genetic diversity in the modern horse illustrated from genome‐wide SNP data. PLoS One, 8, e54997. 10.1371/journal.pone.0054997 23383025 PMC3559798

[ece311197-bib-0032] Petersen, J. L. , Mickelson, J. R. , Rendahl, A. K. , Valberg, S. J. , Andersson, L. S. , Axelsson, J. , Bailey, E. , Bannasch, D. , Binns, M. M. , Borges, A. S. , Brama, P. , da Câmara Machado, A. , Capomaccio, S. , Cappelli, K. , Cothran, E. G. , Distl, O. , Fox‐Clipsham, L. , Graves, K. T. , Guérin, G. , … McCue, M. E. (2013). Genome‐wide analysis reveals selection for important traits in domestic horse breeds. PLoS Genetics, 9, e1003211. 10.1371/journal.pgen.1003211 23349635 PMC3547851

[ece311197-bib-0033] Quan, J. , Cai, Y. , Yang, T. , Ge, Q. , Jiao, T. , & Zhao, S. (2020). Phylogeny and conservation priority assessment of Asian domestic chicken genetic resources. Global Ecology and Conservation, 22, e00944. 10.1016/J.GECCO.2020.E00944

[ece311197-bib-0034] Rout, P. K. , Joshi, M. B. , Mandal, A. , Laloe, D. , Singh, L. , & Thangaraj, K. (2008). Microsatellite‐based phylogeny of Indian domestic goats. BMC Genetics, 9, 1–11. 10.1186/1471-2156-9-11/FIGURES/5 18226239 PMC2268706

[ece311197-bib-0035] Schierup, M. H. , & Hein, J. (2000). Consequences of recombination on traditional phylogenetic analysis. Genetics, 156, 879–891. 11014833 10.1093/genetics/156.2.879 PMC1461297

[ece311197-bib-0036] Sponenberg, P. (1994). Sponenberg evaluation of Roosevelt National Park horses . National Park Service.

[ece311197-bib-0037] Stamatakis, A. (2014). RAxML version 8: A tool for phylogenetic analysis and post‐analysis of large phylogenies. Bioinformatics, 30, 1312–1313. 10.1093/BIOINFORMATICS/BTU033 24451623 PMC3998144

[ece311197-bib-0038] Sun, T. , Huang, G. , Sun, J. , Wang, Z. , Teng, S. , Cao, Y. , Hanif, Q. , Chen, N. , Lei, C. , & Liao, Y. (2020). Mitogenome diversity and maternal origins of Guangxi Buffalo breeds. Animals, 10, 547. 10.3390/ANI10040547 32218165 PMC7222400

[ece311197-bib-0039] Thompson, J. D. , Higgins, D. G. , & Gibson, T. J. (1994). CLUSTAL W: Improving the sensitivity of progressive multiple sequence alignment through sequence weighting, position‐specific gap penalties and weight matrix choice. Nucleic Acids Research, 22, 4673–4680. 7984417 10.1093/nar/22.22.4673 PMC308517

[ece311197-bib-0040] Tollett, C. M. (2018). Genomic diversity and origins of the feral horses (Equus ferus caballus) of Sable Island and the Alberta foothills. University of Saskatchewan.

[ece311197-bib-0041] Vilà, C. , Leonard, J. A. , Götherström, A. , Marklund, S. , Sandberg, K. , Lidén, K. , Wayne, R. K. , & Ellegren, H. (2001). Widespread origins of domestic horse lineages. Science, 291, 474–477. 10.1126/SCIENCE.291.5503.474 11161199

[ece311197-bib-0042] Vonholdt, B. M. , Pollinger, J. P. , Lohmueller, K. E. , Han, E. , Parker, H. G. , Quignon, P. , Degenhardt, J. D. , Boyko, A. R. , Earl, D. A. , Auton, A. , Reynolds, A. , Bryc, K. , Brisbin, A. , Knowles, J. C. , Mosher, D. S. , Spady, T. C. , Elkahloun, A. , Geffen, E. , Pilot, M. , … Wayne, R. K. (2010). Genome‐wide SNP and haplotype analyses reveal a rich history underlying dog domestication. Nature, 464, 898–902. 10.1038/nature08837 20237475 PMC3494089

[ece311197-bib-0043] Wade, C. M. , Giulotto, E. , Sigurdsson, S. , Zoli, M. , Gnerre, S. , Imsland, F. , Lear, T. L. , Adelson, D. L. , Bailey, E. , Bellone, R. R. , Blöcker, H. , Distl, O. , Edgar, R. C. , Garber, M. , Leeb, T. , Mauceli, E. , MacLeod, J. N. , Penedo, M. C. T. , Raison, J. M. , … Lindblad‐Toh, K. (2009). Genome sequence, comparative analysis, and population genetics of the domestic horse. Science, 326(5954), 865–867. 10.1126/science.1178158 19892987 PMC3785132

